# Pre-hospital ECG for acute coronary syndrome in urban India: A cost-effectiveness analysis

**DOI:** 10.1186/1471-2261-10-13

**Published:** 2010-03-12

**Authors:** Joshua Schulman-Marcus, Dorairaj Prabhakaran, Thomas A Gaziano

**Affiliations:** 1 Mount Sinai School of Medicine, One Gustave L Levy Place, New York, NY 10029, USA; 2 Department of Cardiology, All-India Institute of Medical Sciences, New Delhi, India; 3 Division of Cardiovascular Medicine, Brigham and Women's Hospital, Harvard Medical School, USA; 4 Department of Health Policy and Management, Harvard School of Public Health, Boston, MA 02115 USA

## Abstract

**Background:**

Patients with acute coronary syndrome (ACS) in India have increased pre-hospital delay and low rates of thrombolytic reperfusion. Use of ECG could reduce pre-hospital delay among patients who first present to a general practitioner (GP). We assessed whether performing ECG on patients with acute chest pain would improve long-term outcomes and be cost-effective.

**Methods:**

We created a Markov model of urban Indian patients presenting to a GP with acute chest pain to compare a GP's performing an ECG versus not performing one. Variables describing the accuracy of a GP's referral decision in chest pain and ACS, ACS treatment patterns, the effectiveness of thrombolytic reperfusion, and costs were derived from Indian data where available and other developed world studies. The model was used to estimate the incremental cost-effectiveness ratio (ICER) of the intervention in 2007 US dollars per quality adjusted life years (QALY) gained.

**Results:**

Under baseline assumptions, the ECG strategy cost an additional $12.65 per QALY gained compared to no ECG. Sensitivity analyses around the cost of the ECG, cost of thrombolytic, and referral accuracy of the GP yielded ICERs for the ECG strategy ranging between cost-saving and $1124/QALY. All results indicated the intervention is cost-effective under current World Health Organization recommendations.

**Conclusions:**

While direct presentation to the hospital with acute chest pain is preferable, in urban Indian patients presenting first to a GP, an ECG performed by the GP is a cost-effective strategy to reduce disability and mortality. This strategy should be clinically studied and considered until improved emergency transport services are available.

## Background

Ischemic heart disease is already the leading cause of mortality in India [1], and the magnitude of this disease's impact is expected to grow over the next two decades [2]. It is projected that ischemic heart disease will result in two and one-half million Indian deaths by 2020 [3]. Acute coronary syndrome (ACS), including both ST-elevation myocardial infarction (STEMI) and non-ST elevation ACS (NSTE-ACS), is an important manifestation of ischemic heart disease. Rapid diagnosis and treatment with appropriate reperfusion therapies has been proven to increase survival for patients with STEMI. This benefit of reperfusion diminishes as the interval from time of symptom onset to initiation of therapy increases [4]. Current ACS guidelines emphasize the importance of rapid hospital care, especially for STEMI patients who may be eligible for thrombolytic reperfusion within the first twelve hours [4,5].

A recent multi-center Indian registry found only a mean of 58.5% of Indian STEMI patients received thrombolytics, (6% of eligible patients undergo percutaneous revascularization) with an average interval between symptom onset and hospital arrival (pre-hospital delay) of five hours [6]. This was twice as long as the median delay seen in the second Euro Heart survey [7]. Increased pre-hospital delay in India has been attributed to poor patient knowledge about ACS, lack of emergency medical services (EMS) infrastructure, and transportation difficulties [1,6,8,9].

In developed countries, pre-hospital electrocardiography (ECG) performed by EMS technicians is associated with faster access to reperfusion therapies for STEMI patients [10]. As urban EMS systems are often lacking in India, ACS patients have been reported as likely to first present to a general practitioner (GP), which has generally been associated with increased pre-hospital delay [8,9]. However, one retrospective Indian study of hospitalized ACS patients observed that although overall pre-hospital presentation to a GP doubled the risk of significant pre-hospital delay [8], a subgroup in which the GP performed an electrocardiogram (ECG) had reduced delay compared to patients who did not have an ECG and even to those who presented to the hospital directly. This finding was attributed to improved diagnosis of ACS and more prompt referral of patients to a hospital (unpublished data - with permission from Dr. Rajagopalan 5/25/08). These data were obtained under current urban transportation conditions.

It is therefore plausible that a pre-hospital ECG performed by a GP will have an analogous effect in increasing timely access to reperfusion through quicker and more accurate referral to a hospital. Such a strategy could be useful until improvements are made to India's EMS infrastructure. We modeled the hypothesis that compared to an urban GP not performing an ECG, a GP performing one leads to decreased pre-hospital delay, and consequentially increased eligibility for thrombolytics and improved long-term outcomes. Subsequently, we assessed the cost-effectiveness of this ECG strategy compared to not performing one.

## Methods

### Decision-Analytic Model

**Figure 1:**
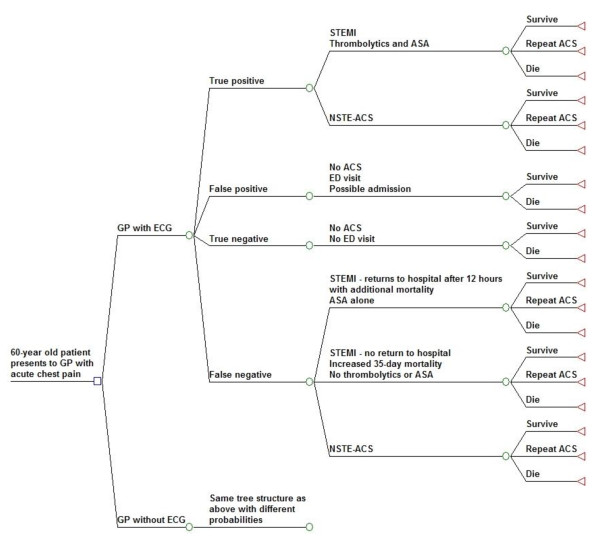
**Markov model (simplified)**. "True positive," "false positive," "true negative," and "false negative" describe the general practitioner's referral decision, and the values are based both on the reported test characteristics as well as the prevalence of acute coronary syndrome (ACS). ASA = aspirin, NSTE-ACS = non ST-elevation acute coronary syndrome, STEMI = ST-elevation myocardial infarction.

We developed a Markov model of urban Indian adult patients presenting to a general practitioner with acute chest pain to assess the overall benefits and costs of the GP performing an ECG versus not performing one (Figure 1). Based on a 2% incidence rate of chest pain this represents about 8 million patients per year in urban areas in India. The model essentially outlined the survival of patients presenting with chest pain. One influence on survival was receiving appropriate thrombolytics for patients with STEMI. This was improved by both increasing the number of correct referrals from the GP and reducing the delay in presentation to a hospital where thrombolytics could be administered. Another influence on improved mortality was modeled for those with NSTE-ACS who are appropriately admitted to the hospital. Costs were also limited by correct referrals. In the ECG arm of the model, we evaluated the referral decision made by GPs based on clinical history, physical examination, and the ECG. In the other arm, the GP's decision was modeled as based on history and physical examination alone. The cohort was modeled until death, either from cardiovascular disease or from other causes with repeated annual cycles. Updates in risk each year were based on age, ACS history and life-tables for India.

Costs and quality adjusted survival were calculated based on estimates derived from prior studies of a GP's accuracy in referring patients with chest pain to either an appropriate medical facility or home. In both arms, we modeled that STEMI patients incorrectly sent home (false negatives) would be delayed in presenting to a hospital, and consequently would be ineligible for thrombolytic treatment. Patients incorrectly referred to a hospital without ACS (false positives) would incur costs of further medical evaluation. We modeled a cohort of adult patients with the median age of sixty years old, which is the median age of ACS in India [6].

In order to model the clinical effect of the GP performing an ECG, we treated the GP's referral decision as a diagnostic test (Table 1). Sensitivity was the fraction of total patients with ACS who were correctly referred, while specificity was the fraction of total patients without ACS who were correctly sent home by the GP. The baseline sensitivities were derived from published evidence [11-16] with minor adjustments from the Indian observations of Rajagopalan and colleagues (unpublished data).

**Table 1:** Baseline values for input variables and costs

	Baseline Value	Source(s)
**Input variables**		
Chest pain is caused by ACS	0.1	[19-22]
GP Sensitivity with ECG	0.818	Unpublished data from [8,11-16]
GP Sensitivity without ECG	0.667	Unpublished data from [8]
GP Specificity with ECG	0.5	[12-16]
GP Specificity without ECG	0.3	[17]
Correctly referred STEMI patients receiving thrombolysis (%)	58.5	[6]
Relative risk reduction of thrombolytics	0.750	[23,24]
		
**Costs (2007 US$)**		
GP visit	1.76	[37]
ECG	1.93	[39,40,42-44]
Emergency department visit	3.48	[37]
Streptokinase	117.00	[38,45]
Admission	157.55	[37]
Blood transfusion	107.67	[37]
Stroke	211.37	[37]
Annual secondary prevention	16.56	[37,38]

ACS = acute coronary syndrome, ECG = electrocardiogram, GP = general practitioner, STEMI = ST-elevation myocardial infarction

Admission costs are for a 5-day admission. The cost of a blood transfusion includes an 3 additional days of hospitalization. The cost of a stroke includes 7 additional days of hospitalization. Annual secondary prevention includes two clinic visits and medications.

Specificity of the GP's referral decision was derived from studies performed in developed countries, as no Indian data were available. The specificity of the referral decision when an ECG was administered was estimated from the approximate mean specificity of admission decisions for patients with acute chest pain in the emergency department [12-16]. The specificity of the decision without an ECG was lower owing to decreased diagnostic certainty based on a study of physicians diagnosing acute myocardial infarction without the use of an ECG [17]. We modeled among patients without ACS incorrectly referred to the hospital by the GP (false positives), a fraction equal to one minus the specificity of GP with the ECG would be admitted for 24 hours to "rule-out" ACS.

We adhered to recommendations of the US Panel on Cost-Effectiveness in Health and Medicine in choosing a societal perspective for the baseline analysis [18]. The entire chest pain cohort was modeled until death, either from cardiovascular disease or other causes. No ethics approval was required, because no patients were used for data collection. All analyses were performed with TreeAge Pro Suite 2008 (TreeAge Software Inc., Williamstown, MA). All authors had full access to all the data and had final responsibility for the decision to submit for publication.

### Chest Pain and ACS Data

ACS is the diagnosis in 10-20% of acute chest pain patients in developed world studies [19-22]. This percentage is unknown in India, so we elected to use the lower bound of 10% for our baseline analysis. This decision was based on the knowledge that the overall prevalence of coronary disease is likely lower and the prevalence of other causes of acute chest pain (e.g. tuberculosis) likely greater compared to developed countries. However, we tested the entire range of values in the sensitivity analysis. Based on the recent CREATE registry, which is the largest reported source of ACS data specific to urban India, we modeled 60.6% as having STEMI and the remainder having NSTE-ACS [6].

The model assumed that 58.5% of patients with STEMI correctly referred by the GP received thrombolytics, which is the Indian average [6]. As Rajagopalan and colleagues observed a median delay of 24 hours for ACS patients who were misdiagnosed by a GP (unpublished data), we modeled that none of the STEMI patients incorrectly sent home by the GP received thrombolytics regardless of eventual hospitalization status. The short- and long-term effectiveness and risks of thrombolytics were derived from the overall effects reported in the Second International Study of Infarct Survival (ISIS-2) and Fibrinolytic Therapy Trialists (FTT) studies [23-25]. All patients who were treated in a hospital received early aspirin therapy, which had a relative risk reduction derived from ISIS-2.

Mortality to 35 days was estimated from ISIS-2 for STEMI patients [23,24], and from the CREATE registry for NSTE-ACS patients [6]. Patients with STEMI who were incorrectly sent home by the GP but later returned to the hospital were modeled to have a twelve-hour pre-hospital delay conveying an additional 0.9% mortality, which is half the first-day STEMI mortality rate in the FTT and Clopidogrel and Metoprolol in Myocardial Infarction (COMMIT) studies [25,26]. All ACS patients who were incorrectly sent home by the GP and did not return to the hospital had double the 35-day mortality of those admitted, a hazard determined from developed world studies of ACS patients incorrectly sent home from the emergency department [11,21].

Mortality after 35 days for chest pain patients without ACS was derived from World Health Organization (WHO) life tables for India [27]. We assumed that patients without ACS did not have ischemic heart disease, so we adjusted the annual mortality probabilities to decrease the influence of heart disease for the first ten years. In the patients who had ACS, for the first ten years we used mortality and reinfarction data derived from Law and colleagues [28], with adjustments for the effects of secondary prevention. After ten years, the annual mortality for ACS patients was equivalent to the sum of fatal reinfarction and the yearly mortality in the WHO life tables.

For secondary prevention after 35 days, we considered a regimen of aspirin, a beta-blocker, a statin, and an angiotensin converting enzyme (ACE) inhibitor. As an international survey showed that the actual population of patients taking secondary prevention in India is well below those eligible [29], we modeled the relative risk reduction of secondary prevention only for the percentage of Indian patients taking the individual drugs. The relative risk reductions of the secondary prevention regimen were derived from the reported literature [30-33]. In accordance with evidence that has suggested a diminishing effect of beta-blockers over time [34], the beta-blocker was only included in the secondary prevention regimen for the first six years of the model. We assumed that the relative risk reductions of the drugs were independent, and therefore we calculated the overall effect by multiplying the individual relative risks associated with each drug.

### Outcomes and Costs

Outcomes in the analyses were measured in quality adjusted life-years (QALYs) gained and net health costs. QALYs were obtained by using the disability weights of the WHO Global Burden of Disease Project as used in the 2nd edition of the Disease Control Priorities (DCP) Project [35,36].

Costs of hospital admission, salaries, office visits, and laboratory fees were derived from regional estimates done for the Disease Control Priorities Project [37]. As the median duration of hospitalization for ACS in India is five days [6], we modeled a five day admission to a secondary hospital with the pro-rated labor costs of a cardiologist, junior doctor, nursing care, and an administrative worker. For patients who needed a blood transfusion or stroke care owing to the adverse effects of thrombolytics, additional days of hospitalization were added. Drug costs were calculated from the International Drug Price Indicator Guide [38] and local costing data where available. All secondary prevention drug costs were computed as a year's supply, though covering only the proportion of Indian patients taking medical therapy as reported in the WHO-PREMISE survey [29].

The baseline cost of an ECG was estimated as the current reimbursement rate to private hospitals by India's Central Government Health Scheme (CGHS) in large metropolitan areas [39]. An additional $0.08 cost was added based on a 500 rupee training cost [40] spread over an estimated 30 annual ECGs performed over a five year period. We did not model the direct costs of transportation to the hospital, time lost from work, or rehabilitation. Initial costs obtained in India rupees prior to 2007 were inflated to 2007 rupees using the Indian wholesale price index [41] and then converted to 2007 US dollars with the midyear exchange rate. The costs are modest but the Gross National Income (GNI) per capita was approximately $800 in 2006, reflecting the relatively cheap labor supply in India and as a result lower costs.

All costs and benefits were tallied at mid-cycle increments. Those dying during a cycle were assumed to incur one half of the utilities and costs of that cycle. All costs as well as health outcomes were discounted at 3% per year, which is consistent with guidelines [18]. Incremental cost-effectiveness ratios (ICERs) were calculated as the difference in costs between the ECG intervention and no intervention divided by the increase in QALYs gained.

### Sensitivity Analysis

One-way sensitivity analyses on the "sensitivity" and "specificity" of the GP's referral decision relied on studies of admission decisions for emergency department patients with acute chest pain [12-16]. Analyses of the effectiveness of the thrombolytics relied on the standard deviations reported in ISIS-2. As the efficacy of therapy in real Indian settings may be substantially lower than that in developed world controlled trials, we also assessed reduction in thrombolytic efficacy of up to 50%. Sensitivity analyses of the costs of health-care delivery relied on the ranges reported in the DCP working paper [37], except for the cost of an ECG which was estimated from selected Indian price schedules as well as one developed world reimbursement schedule for comparison [42-44]. Analysis of the price of thrombolytic relied on a lower bound from an online Indian database of drug prices [45] and an upper bound from the International Drug Price Indicator Guide. In our sensitivity analyses, we use the term "cost-saving" to mean that the ECG intervention cost less and resulted in more QALYs gained than not performing an ECG. We also performed a probabilistic sensitivity analysis[46] with second-order Monte Carlo simulation of 1000 randomly selected sets of parameters in which we simultaneously sampled values from the distributions with corresponding logit-means and logit-standard deviations.

## Results

Under baseline assumptions, the strategy in which a GP did not perform an ECG resulted in 12.423 QALYs obtained over an average lifetime, while performing an ECG resulted in 12.435 QALYs obtained (Table 2). Approximately 57% of this gain in QALYs came from the increased use of appropriate thrombolytics, 30% from increased use of secondary prevention treatment and the remaining 12% was related to improved hospital survival for those appropriately referred as a result of the ECG. The strategy of not performing an ECG cost $50.37 per individual over the lifetime of the cohort, while the ECG intervention cost $50.52 per individual. Overall, the ECG intervention had an incremental cost-effectiveness ratio (ICER) of $12.65 per QALY gained.

**Table 2:** Results with baseline assumptions

	Cost (2007 US$)	Effects (QALYs obtained)	ICER ($/QALY gained)
GP without ECG	50.37	12.423	
GP with ECG	50.52	12.435	
			
Incremental Change	0.15	0.012	12.65

ECG = electrocardiogram, GP = general practitioner, ICER = Incremental cost-effectiveness ratio, QALY = quality adjusted life year

One-way sensitivity analyses around the "sensitivity" and "specificity" of the GP's referral decisions resulted in ICERs that ranged between cost-saving to less than $400/QALY (Table 3). The model was largely insensitive to changes in the referral accuracy (sensitivity) of the GP with an ECG, yielding ICERs from cost-saving to $103/QALY. When the sensitivity of the GP without an ECG was set to be equal to the baseline value of the GP with the ECG, while holding the specificity constant, the ECG intervention was cost-saving. When the same test was performed for the specificity of the GP without an ECG while holding the sensitivity constant, the ICER was $351/QALY. If the sensitivity of the GP with ECG was lower than the GP without an ECG then the no ECG strategy could have a ICER of $580/QALY when compared to the GP with ECG.

**Table 3:** Results of one-way sensitivity analysis

	Baseline Value	Tested Range	ICER* ($/QALY gained)
**Input variables**			
Chest pain is caused by ACS	0.1	0.01 -- 0.2	Cost-saving† -- 119
GP sensitivity with ECG	0.818	0.7 -- 0.98	Cost-saving -- 103
GP sensitivity without ECG	0.667	0.667 -- 0.818	13 -- cost-saving
GP specificity with ECG	0.5	0.44 -- 0.67	76 -- cost-saving
GP specificity without ECG	0.3	0.3 -- 0.5	57 -- 351
Relative risk reduction of thrombolytics	0.750	0.71 -- 0.875	13 -- 12
			
**Costs (2007 US$)**			
ECG	1.85	1.00 -- 15.00	Cost-saving -- 1124
Streptokinase	117.00	68.74 -- 162.38	Cost-saving -- 33
Admission	157.55	99.71 -- 459.39	Cost-saving -- 73

* The ICER is a comparison of the GP with ECG compared to GP without an ECG, with the value on the left corresponding with the leftward most value in the tested range. All ICERs are rounded to the nearest dollar.

† "Cost-saving" here is defined as the ECG intervention costing less and increasing QALYs gained compared to no ECG.

ACS = acute coronary syndrome, ECG = electrocardiogram, ICER = incremental cost-effectiveness ratio, QALY = quality adjusted life year

**Figure 2:**
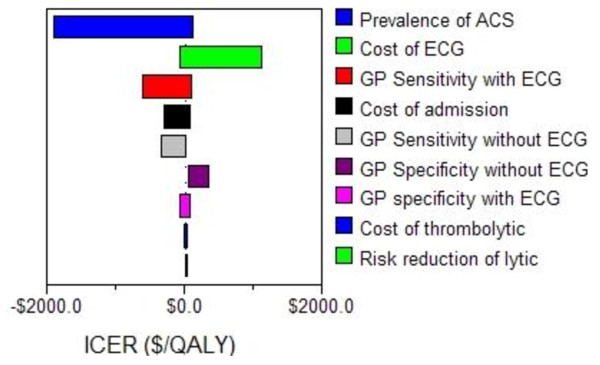
**Tornado diagram**. This Tornado diagram shows the incremental cost-effectiveness ratio (ICER) of the range of values for each variable tested in the one-way sensitivity analysis. A negative ICER is described in the text as "cost-saving."

Sensitivity analysis around the prevalence of ACS resulted in an ICER that ranged from cost-saving to $119/QALY (Figure 2). If the ECG's cost was modeled as the maximum Indian cost of approximately $4, the ICER of the ECG strategy was approximately $189/QALY. When the ECG cost $15, about the procedure's cost in the United States [44], the ECG intervention yielded an ICER of $1124/QALY. The threshold at which the ICER for the cost of the ECG changed from cost-saving to a positive value (cost more and gained more QALYs) was approximately $2. Sensitivity analyses on the cost of admission and efficacy of the thrombolytic resulted in minor variation in the baseline ICER.

We performed a probabilistic sensitivity analysis (PSA) on the main variables of uncertainty, including the sensitivity and specificity, the efficacy of thrombolytics in India, the cost of ECGs in India. The PSA using the variables in Table 3 resulted in a mean ICER of $154/QALY (95% confidence interval, -$1790/QALY., $1830/QALY). Greater than 97.5% of the values were less than a willingness to pay threshold of $3000.

## Discussion and Conclusions

We modeled a cohort of urban Indian patients with acute chest pain presenting to a general practitioner to compare the lifelong cost-effectiveness of a GP performing an ECG versus not performing one. Our model found that it cost only $12.65 to gain an additional quality adjusted life year for this cohort. The relatively cheap cost for a life year results from the ECG facilitating a GP's decision in the early and appropriate referral of STEMI to a hospital where thrombolysis is administered, as well as a reduction in unnecessary referrals of patients without ACS to hospitals in order to "rule-out" an event. One-way sensitivity analyses around uncertain variables and assumptions demonstrated that this finding is robust, especially for the values most commonly found in contemporary Indian settings.

WHO considers interventions to be cost-effective if they have ICERs that are less than three times gross national income (GNI) per head [47]. In 2006, India's GNI per capita was $820, or $2460 after adjustment for purchasing power parity [48]. As the baseline findings of our model and all sensitivity analyses suggest the ICER of the GP's performing an ECG compared to not performing one is under $1200, the ECG strategy is certainly cost-effective under current WHO definitions. Furthermore, the results of the probabilistic sensitivity analyses suggest the findings are robust with a 95% confidence interval that is well below the likely willingness to pay threshold for India based on WHO recommendations. Also, since we did not model the direct costs of missed work owing to unnecessary hospitalization of chest pain patients without ACS, it is likely that the ECG strategy is even more cost-effective than our results indicate.

Current guidelines recommend that all patients with acute chest pain should be educated to directly present to a hospital emergency department, and that any such patients presenting to an outpatient practitioner should be urgently referred [4]. We did not model a direct presentation strategy in this study owing to inadequate data, though we think it would be interesting to compare its cost-effectiveness to pre-hospital GP strategies in the future. While such a direct presentation strategy is likely to further increase the population eligible for thrombolysis, it may also cost substantially more owing to increased chest pain patients without ACS presenting to crowded emergency rooms. Still, in the absence of further evidence, we emphasize that direct presentation with acute chest pain is preferable.

In the meantime, while the Indian public undergoes public education regarding the association of chest pain with serious conditions such as myocardial ischemia and the possibility of myocardial infarction and the need to present to an appropriate facility in a timely fashion; the use of ECG machines by general practitioners may facilitate a timely presentation to an appropriate hospital where acute treatments such as thrombolytics can be applied. Training to use the ECG machine and interpret results can be obtained for as little as $10 and machines can be purchased for under $300. Many private based GPs already may have them. Government based clinics could consider their purchase if not already available. The policy of using ECG machines may not be feasible in rural areas however, given the access to facilities where thrombolysis can be safely be administered may not available.

We did not model a strategy whereby a GP would use a point-of-care troponin assay to make a referral decision. Troponins are frequently negative early in ACS [4] and it is unclear how useful a pre-hospital troponin assay would be when compared to or combined with an ECG. A pre-hospital troponin test's impact on mortality is also uncertain, as troponin assays mostly increase the sensitivity for NSTE-ACS [49], for which thrombolytics are not indicated. We think this is an area that deserves additional research

Sensitivity analyses around the "sensitivity" and "specificity" of the GP's referral decisions with and without the ECG robustly support our baseline findings. We performed these analyses using an especially broad range of possible values for two reasons. First, most data regarding physicians' performance in the diagnosis and referral of ACS patients using ECG are from emergency departments; it is unknown if outpatient GPs perform similarly. Second, there is very limited knowledge about how real physicians perform in the diagnosis and referral of patients with ACS when there is no ECG available. Therefore, in the sensitivity analysis of the GP's referral decision without the ECG, we extended the tested range of values up to the baseline values for the GP with the ECG. This is a very conservative assumption, for it is the equivalent of stating that the ECG is not helpful in referral decision-making, which is at odds with published guidelines [4]. Even so, these one-way analyses did not greatly affect the calculated cost-effectiveness of the ECG strategy

There are several limitations to our study. First, our baseline estimates of the "sensitivity" of a GP's referral decision are based on supplementary observations of a small single-center study that had of mainly urban middle-class patients with confirmed ACS [8]. No confidence intervals were reported in that study. The primary study itself was retrospective, and it was not designed to evaluate a GP's pre-hospital decision making. However, these are the only Indian data available studying a scenario common to urban India. Sensitivity analysis using a wide range of values derived from developed world studies for the sensitivity of GP's referral decision demonstrates that our findings are robust.

Second, owing to limited data on the ultimate diagnosis of acute chest pain across different age groups in India, we only modeled a cohort of sixty-year old patients. We assumed that ACS was the cause of chest pain in 10% of the cohort, which was the lower end of the reported range in developed world studies [19-22]. However, sensitivity analysis supports our finding that the ECG strategy is cost-effective throughout the entire reported range. We found that when ACS as a cause of chest pain was less prevalent, as might be expected in younger cohorts, the ECG strategy was actually cost-saving. When ACS was the cause of acute chest pain in 20% of the modeled cohort, which may be true in older cohorts of patients, the ECG strategy still had an ICER still well beneath the WHO threshold for cost-effectiveness. Further, a previous study has shown that the use of thrombolytics in the elderly led to an cost-effectiveness ratio for those over the age of 75 what was double of that for those under the age of 70[50] Given that women present at a later age and over all with lower prevalence this would tend to make the ratios in general lower for women than men, especially since case-fatality is higher for females.

Third, owing to limited Indian data, our model did not study the hour-dependent differential effects of thrombolytics on mortality. Major international studies suggest that the subgroup receiving thrombolytics within three hours of ACS onset benefits more than the subgroups receiving them from 3-6 and 6-12 hours after onset [23,25]. Our model treated receiving thrombolytics as a binary variable, and the risk reduction in mortality was the average reported from a large international trial. We predict that modeling thrombolytic administration as a time-dynamic variable would lead to even greater reductions in mortality, which would further favor the ECG strategy. This is a topic that deserves further research once Indian data are available.

A recent study in the United States suggests that pre-hospital ECG performed by emergency medical services increase the frequency and rapidity of reperfusion therapies for patients with STEMI [10]. Given that many Indian ACS patients present first to a GP and the current absence of emergency systems, it is reasonable to study whether a GP performing pre-hospital ECG could yield analogous benefits. We modeled such a GP-based strategy and found it to be robustly cost-effective compared to a GP not performing an ECG for urban patients with acute chest pain. This finding should be considered a strong rationale for clinical trials. Meanwhile, our findings also suggest that it is sensible for GPs in urban India to perform an ECG in patients with acute chest pain until improvements to India's emergency medical services are put in place.

## Competing interests

The authors declare that they have no competing interests.

## Sources of support

Sarnoff Cardiovascular Research Foundation (J Schulman-Marcus) Fogarty International Center, National Institutes of Health: K01TW007141-01 (TA Gaziano)

## Authors' contributions

JSM and TAG - study conception; study design; data collection, analysis, and interpretation; drafting manuscript; decision to publish. DP - data provision; data analysis and interpretation; drafting manuscript; decision to publish. All authors read and approved the final manuscript.

## Pre-publication history

The pre-publication history for this paper can be accessed here:

http://www.biomedcentral.com/1471-2261/10/13/prepub
